# Electroacupuncture for Spinal Cord Injury: A Systematic Review and Meta-Analysis of Randomised Controlled Trials

**DOI:** 10.1155/2022/8040555

**Published:** 2022-03-04

**Authors:** Jiuqing Tan, Fangqi Meng, Baobao Zhang, Qingwen Deng, Boyu Jiao, Lizhi Peng, Ying Ding, Jingwen Ruan, Jingchun Zeng, Wenya Pei, Guohua Lin

**Affiliations:** ^1^The First Affiliated Hospital, Guangzhou University of Chinese Medicine, 16 Airport Road, Guangzhou 510405, Guangdong, China; ^2^The First Affiliated Hospital, Sun Yat-sen University, 58 Zhongshan 2nd Road, Guangzhou 510080, Guangdong, China; ^3^Zhongshan School of Medicine, Sun Yat-sen University, 74 Zhongshan 2nd Road, Guangzhou 510080, Guangdong, China; ^4^Shenzhen Nanshan District Hospital of Traditional Chinese Medicine, Shenzhen Bay Section 2248-8 Shekou Street Zhongxin Road, Shenzhen 518054, Guangdong, China

## Abstract

**Background:**

Previous studies have shown that electroacupuncture (EA) has a positive effect on motor and sensory function in patients with spinal cord injury (SCI). This review evaluated the effectiveness of EA for improvement in activities of daily living in patients with SCI.

**Methods:**

We searched the Cochrane Library, PubMed, Web of Science, CNKI, WanFang Data, and VIP databases using a search strategy according to the guidelines of the Cochrane Handbook for Systematic Review of Interventions up to 30th September 2020. Only randomized controlled trials (RCTs) of EA in patients with SCI were included. We analyzed the data using RevMan (version 5.3) and graded the quality of evidence using GRADE profiler 3.6.1.

**Results:**

This meta-analysis included 10 RCTs with 712 patients. Three studies revealed that the functional independence measure score for SCI patients in the EA group was higher than that in the control group (mean difference [MD] = 13.46, 95% CI: 8.00 to 18.92, *P* < 0.00001). Five studies showed that the modified Barthel index in the EA group was higher than that in the control group (MD = 6.92, 95% CI: 4.96 to 8.89, *P* < 0.00001). Five studies showed that the American Spinal Injury Association-motor score (ASIA-motor score) in the EA group was higher than that in the control group (standard MD = 0.96, 95% CI: 0.75 to 1.18, *P* < 0.00001). Three studies reported the ASIA-tactile and pain scores and also reported that the scores in the EA group were higher than those in the control group, with high homogeneity (tactile I^2^ = 86%, *P* = 0.0008; pain I^2^ = 54%, *P* = 0.11). The quality of evidence for the use of EA for improvement in motor and sensory function in SCIs was moderate according to the GRADE system.

**Conclusion:**

This review suggested that EA improves activities of daily living and motor function in patients with SCI, with a moderate level of evidence.

## 1. Introduction

Spinal cord injury (SCI), which can be caused by many factors, such as trauma, inflammation, and tumor compression, leads to partial or complete loss of sensory and motor functions below the injured segment and even loss of respiratory function. According to a global epidemiological survey, SCIs from accidents are increasing, and approximately 500,000 cases of SCIs worldwide occur every year [1, 2]. Due to the complex pathophysiology of SCI, spontaneous repair or regeneration of the nerve cells in the injured segments is less probable. Therefore, restoring the function of damaged nerves is challenging. Patients with complete SCI require long-term bed care, which increases the burden on the family and society [3].

At present, there are many clinical treatments for SCI, such as timely decompression [4, 5], methylprednisolone [6], stem cell transplant [7, 8], nerve growth factor therapy [9, 10], and spinal cord electrical stimulation therapy [11]. Acute spinal cord injury is mainly treated by surgery, which limits the scope of the injury and reduces secondary injury as much as possible; however, functional reconstruction during the recovery period determines the patient's prognosis and quality of life. Although many therapies have been used to treat SCIs, rehabilitation is important for improvement in motor and sensory functions [12, 13]. However, due to the complicated pathological mechanism of SCI, achieving satisfactory clinical effects with these treatment methods remains challenging.

Previous studies have confirmed that electroacupuncture (EA) can improve motor and sensory functions after an SCI [14, 15], including complications such as urinary incontinence [16] and chronic pain [17, 18]. Many experimental studies have reported that EA treatment can increase neurotrophic factor secretion, inhibit inflammation, and promote axonal regeneration, synapse formation, and neural rehabilitation after SCI [19, 20]. However, evidence showed that EA improved motor and sensory functions, as well as the quality of life, remain insufficient, and there is a lack of detailed analysis of the treatment duration and acupoint selection of EA. The present study systematically evaluated the clinical efficacy of EA for motor and sensory functions and activities of daily living (ADLs) in patients with SCI. This article is more inclined to the improvement of ADLs since the current rehabilitation concept pays more attention to the recovery of overall function and even returns to their profession. This study was conducted according to the principles of evidence-based medicine.

## 2. Methods

### 2.1. Protocol and Registration

This review was registered with the International Platform of Registered Systematic Review and Meta-analysis Protocols (INPLASY), with the registration number INPLASY202050033.

### 2.2. Search Strategy

All studies on EA treatment for SCI were included, with no restrictions on language or publishing status. We searched the Cochrane Library, PubMed, Web of Science, and three Chinese databases (CNKI, WanFang Data, and VIP). For details on the search strategy, see the Supplemental information. Our search strategy followed the guidelines outlined in the Cochrane Handbook for Systematic Review of Interventions (version 5.1.0), and we considered studies published until 30 September 2020. Randomized controlled trials (RCTs) assessing the effects of interventions for SCIs were scanned for inclusion in this study. We also scanned quasirandomized and other observational studies that were retrieved alongside the RCTs for reports of adverse effects.

### 2.3. Selection Criteria

#### 2.3.1. Types of Studies

This study included RCTs on the use of EA on patients with SCIs. Human studies that were not RCTs, animal experiments, reviews, dissertations, and case reports were excluded.

#### 2.3.2. Participants/Population

Patients with an SCI were included in the study regardless of their age, sex, or ethnicity.

#### 2.3.3. Interventions

We included studies that used EA alone and those that combined EA with other treatments as the treatment for the experimental group.

#### 2.3.4. Comparators

Blank control, placebo acupuncture, rehabilitation therapy, and medicine served as comparators.

#### 2.3.5. Outcomes

For a study to be included in this review, it should have reported on one or more of the following three main outcome measures: functional independence measure (FIM), modified Barthel index/Barthel index (MBI/BI), or the American Spinal Injury Association (ASIA) impairment scale for patients with SCI. FIM can be used to assess the daily activities of people with various diseases or injuries. The assessment includes six domains, namely self-care activities, sphincter control, transfer, travel, communication, and social cognition. The MBI/BI is one of the most common methods for assessing ADL. It includes the following 10 items: eating, clothing, dressing, bathing, stool control, urination control, toileting, bed and chair transfer, walking on flat ground, and ascending or descending stairs. The ASIA Spinal Cord Injury Assessment Standard assesses motor, pain, and tactile function.

### 2.4. Quality Assessment

Two authors (B. Y. Jiao and Q. W. Deng) conducted independent quality assessments based on the Cochrane Risk of Bias Assessment Tool [21]. The assessment items included the randomization method, allocation concealment, blinding method implementation, outcome indicator completeness, selected outcome reporting, and other risks of bias. Each item was classified as low risk, unclear risk, or high risk. When the evaluation results of the two assessors varied, they discussed the variations to arrive at a consensus.

### 2.5. Data Extraction Analysis

Two authors (B. B. Zhang and F. Q. Meng) independently extracted the following data from each study using Epi Data (version 3.1): (1) bibliographic information of the study including the title, first author, year, language, and country of publication; (2) the inclusion and exclusion criteria; (3) baseline characteristics including the number of study centers, group setting, sample size, gender composition, age, and treatment course; (4) interventions provided for the observation and control groups (methods, duration, frequency, and the main selected acupuncture points); (5) outcome indicators; and (6) the randomization method, allocation concealment, blinding methods, and other methodological information.

### 2.6. Statistical Analysis

We performed the statistical analyses using RevMan (version 5.3). The relative risk (RR) was reported for count data, while mean differences (MD) or standard mean differences (SMD) were reported for continuous variables. If the unit or measurement method for an included variable varied or the range of values was wide, we used the SMD; MD was applied in all other cases.

We assessed statistical heterogeneity between studies using I^2^ and its corresponding *P*-value. *I*^2^ > 50% was considered high heterogeneity, 25%–50% was considered moderate, and <25% was considered low. When *I*^2^ < 50% and *P* > 0.1, we used a fixed-effects model. When *I*^2^ > 50% and *P* < 0.1, we used a random-effects model. Additionally, we performed subgroup analysis for any studies with statistical heterogeneity based on the patient characteristics and interventions for the control groups by adapting their outcome measurement tools and performing sensitivity analysis when necessary.

We used the GRADEpro (version 3.6) to assess the overall quality of evidence. This grading method for each outcome measure assessed the risk of bias, inconsistency of results, indirectness of evidence, imprecision, and publication bias. A *P* value of <0.05 was considered statistically significant.

## 3. Results

### 3.1. Literature Search and Selection

**Figure 1:**
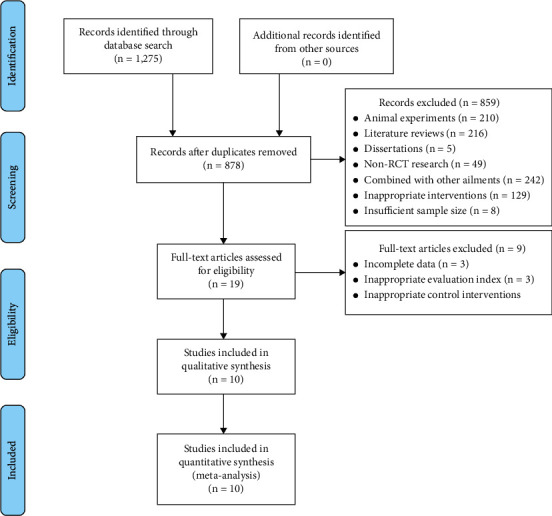
Literature screening process.

We retrieved a total of 1,275 records (1,177 Chinese articles and 98 English articles) through a preliminary search of the databases. After reading the titles, abstracts, and full texts, 10 articles with a total of 712 cases—357 in the EA group and 355 in the control group—were included [22–31]. A flow diagram of the study selection process is shown in Figure 1 (Cohen's kappa = 0.507).

### 3.2. Study Characteristics

We summarised the basic characteristics of the included studies. The demographic characteristics of the included studies are shown in Table 1, and the clinical characteristics are presented in Table 2. The treatment course in each study ranged from 8 weeks to 1 year, and most of the treatment durations were 30 min, five or six times per week. Eight of the studies combined EA with rehabilitation therapy for the intervention group and used only rehabilitation therapy for the control group [22–27, 30, 31]. Five of the ten studies included in this review used Governor Vessel (GV) acupoints as the main acupoints for treating SCIs [23, 24, 26, 28, 30], and one study used *Houxi* as the main therapeutic point [31]. *Jiaji* was the main acupoint in six studies [22, 25, 26, 28–30]. None of the included studies reported any adverse events, which indicates that the safety factor of EA may be high, and EA rarely leads to adverse events.

### 3.3. Assessment of Quality and Bias

**Figure 2:**
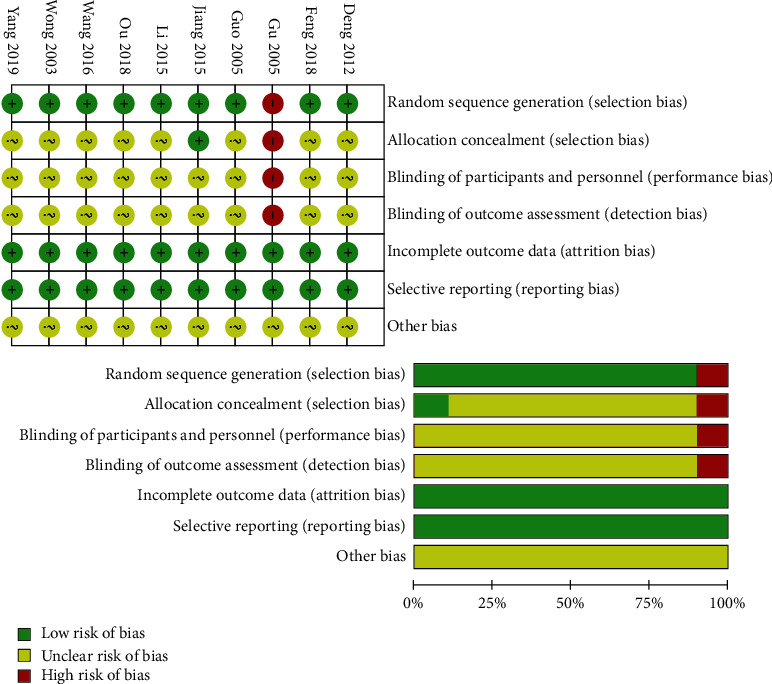
Plots of the risk of bias for the included studies.

The details of the assessment of quality and bias are shown in Figure 2 (Cohen's kappa = 0.703). One study [22] used an invalid randomization method (random grouping according to the number of patients in the hospital); thus, it was assessed as having a high risk of bias. Most of the included studies did not describe the allocation concealment process. As it is difficult to perform blinding in acupuncture experiments, most of the studies also did not discuss the blinding methods.

### 3.4. Outcomes Measures

#### 3.4.1. FIM

**Figure 3:**
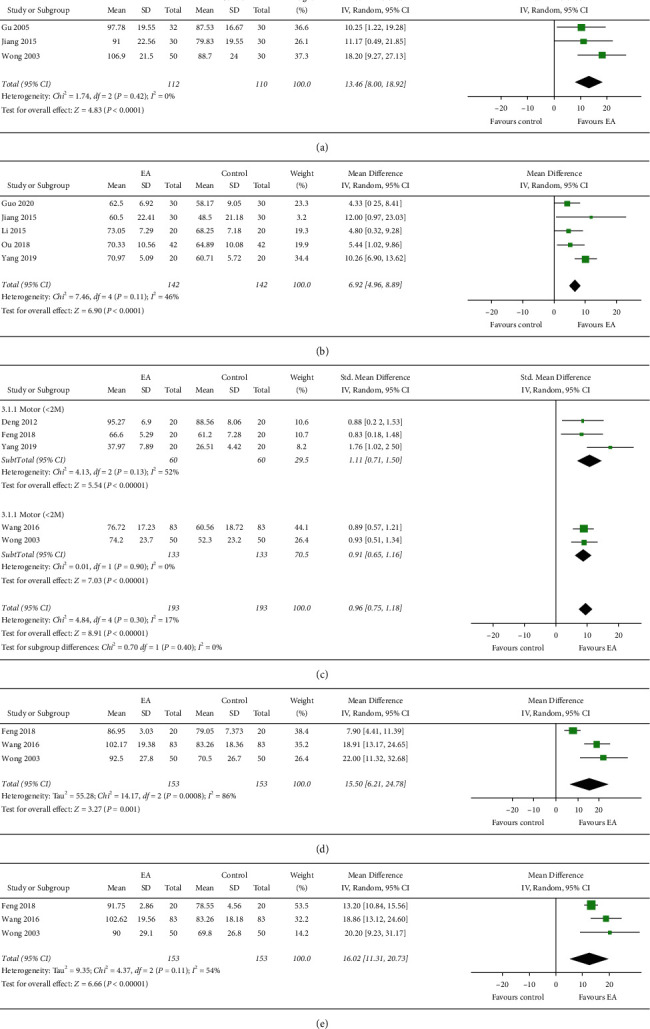
Effect of EA on FIM, MBI/BI, and ASIA scores. (a) Effects on EA on FIM. (b) Effects on EA on MBI/BI. (c) Effects on EA on ASIA-motor score. (d) Effects on ASIA-tactile score. (e) Effects on EA on ASIA-pain score.

Three of the ten studies reported the FIM, with a low homogeneity (I^2^ = 0%, *P* = 0.42 > 0.05). Therefore, we used a fixed-effects model for the calculations. FIM scores were higher in patients with SCI treated with EA than in the controls (MD = 13.46, 95% CI: 8.00 to 18.92, *P* < 0.00001; Figure 3(a)).

#### 3.4.2. MBI/BI

We included five studies with a total of 284 participants that compared the MBI/BI of the EA and control groups. In these studies, the MBI/BI was higher in the EA group than in the control group. Additionally, heterogeneity was moderate between the trials (I^2^ = 46%, *P* = 0.11 > 0.05), and we used a fixed-effects model for the analysis. The MBI/BI of the patients with SCI in the EA group exceeded that of those in the control group (MD = 6.92, 95% CI: 4.96 to 8.89, *P* < 0.00001; Figure 3(b)).

#### 3.4.3. ASIA-Motor Score

Five studies examined the ASIA motor scores. We reported the SMD when there was poor homogeneity (I^2^ = 17%, *P* = 0.30 > 0.05) and used a fixed-effects model for the analysis. Our results showed that the ASIA-motor score of the EA group was higher than that of the control group (SMD = 0.96, 95% CI: 0.75 to 1.18, *P* < 0.00001; Figure 3(c)). We performed subgroup analysis by treatment duration, which indicated a higher score when the treatment duration exceeded 2 months than when the treatment was 2 months or less.

#### 3.4.4. ASIA-Tactile Score

Three studies examined the ASIA-tactile score with high homogeneity (I^2^ = 86%, *P* = 0.0008 < 0.05). Therefore, we used the random-effects model for the analysis. There was a statistically significant difference between the ASIA-tactile score of the EA group and of the control group (MD = 15.50, 95% CI: 6.21 to 24.78, *P* = 0.001; Figure 3(d)).

#### 3.4.5. ASIA-Pain Score

The three studies also examined the ASIA-pain score, with slightly increased homogeneity (I^2^ = 54%, *P* = 0.11 > 0.05), in a random-effects model. The ASIA-pain score of the EA group exceeded that of the control group (MD = 14.25, 95% CI: 12.11 to 16.39, *P* < 0.00001; Figure 3(e)).

### 3.5. Sensitivity Analysis and Publication Bias

In the above meta-analysis, the ASIA-tactile and pain scores showed high heterogeneity, the MBI/BI showed moderate heterogeneity, and the remaining indicators showed low heterogeneity. To explore the source of heterogeneity in the ASIA-tactile score meta, we eliminated the studies one by one and found that when we excluded the study by Feng et al. [23], heterogeneity declined (I^2^ = 0%, *P* = 0.62 > 0.05; Supplemental information, Figure 1). This suggested that the heterogeneity of the ASIA-tactile score was likely derived from Feng et al. [23]. In the ASIA-pain score, the heterogeneity also reduced (I^2^ = 0%, *P* = 0.83 > 0.05 Supplemental information, Figure 2) when we eliminated the study by Feng et al. [23]. Thus, we concluded that the heterogeneity in the ASIA-pain score meta-analysis was derived primarily from Feng et al. [23]. Furthermore, meta-analysis of MBI/BI showed moderate heterogeneity between trials (I^2^ = 46%, *P* = 0.11 > 0.05; Supplemental information, Figure 3). By excluding articles one by one, we found that excluding the study by Yang et al. [30] eliminated the heterogeneity (I^2^ = 0%, *P* = 0.64 > 0.05), implying that their study was probably the source of the heterogeneity. Due to the small number of studies included in this meta-analysis, the use of funnel charts for publication bias analysis would have been of little significance. Therefore, publication bias analysis was not performed in this study.

### 3.6. Evaluating the Evidence

We evaluated the evidence for the above results using the GRADEpro (version 3.6). Our findings showed low- and moderate-level evidence that EA is an effective treatment for SCIs. Due to the absence of specific descriptions of the randomization methods and the implementation of effective blinding methods in the included studies, evidence indicating that EA improves the FIM scores and MBI/BI of patients with SCI was moderate (Supplemental information, Tables 1 and 2). Similarly, the evidence that EA improves the ASIA scores of patients with SCI was downgraded on the “Risk of Bias”. Moreover, ASIA-pain and ASIA-tactile were rated as “serious” and “very serious” for “inconsistency” due to their high heterogeneity, which decreased the quality of the evidence (Supplemental information, Table 3).

## 4. Discussion

### 4.1. Summary of Results

The ten studies which were included in this meta-analysis systematically evaluated the clinical efficacy of EA for motor and sensory function and ADLs in patients with SCI. The meta-analysis results of the FIM and MBI/BI showed that EA could improve ADLs in patients with SCI. In addition, the meta-analysis of the MBI/BI showed mild heterogeneity. After the article by Yang et al. [30] was excluded, the heterogeneity was reduced, and by comparing the five studies, including the study by Yang et al. [30], that reported on the MBI/BI, we discovered that Yang et al. [30] left out important general information, such as sex, age, and disease course, from their RCT. Their study was the only one among the five studies that did not refer to a randomization process. Thus, we considered that the incomplete basic information and the lack of randomization in their study may have contributed to the high-level of heterogeneity observed earlier.

Additionally, a meta-analysis of the ASIA motor score indicated that EA could improve motor function in patients with SCIs. However, although there was a statistically significant difference between the EA group and the control group (*P* < 0.05 in the meta-analysis of ASIA-pain and ASIA-tactile scores), the included studies had high heterogeneity. To explore the reason for the high heterogeneity of several indicators (ASIA-tactile and pain score and MBI/BI), we conducted a sensitivity analysis by excluding studies one by one. The findings showed that excluding the study by Feng et al. [23] decreased the heterogeneity of the ASIA sensory score (tactile and pain), which indicated that Feng et al. [23] was probably the source of the high heterogeneity. Comparing the clinical characteristics of the three RCTs that evaluated this index, we found that the study by Feng et al. [23] had the least number of patients (20/20), the shortest treatment time (8 weeks), the smallest number of acupoint selections, and no description of acupoint matching. These factors may explain the heterogeneity observed before their study was excluded. Furthermore, the subgroup analysis of the ASIA-motor scores according to the treatment duration indicated a higher score when the treatment duration was beyond 2 months than when it was below 2 months. These results suggested that the duration for acupuncture treatment in the clinic to improve the motor function of patients with SCI should be at least two months.

According to the GRADE evidence evaluation, there was moderate evidence that EA can improve ADL-related scores (such as FIM and MBI/BI) and motor function and low evidence that EA can improve sensory function in patients with SCI.

### 4.2. Previous Research

Previous studies have suggested the effectiveness and safety of acupuncture therapy for patients with SCI [32]. Regarding outcome indicators, the studies reported the recovery of motor function and ADLs in patients with SCI but did not report on sensory function. In addition, these studies reported on general acupoints rather than the meridians of the acupoints; thus, these studies did not follow the dialectical theory of acupoint selection. Although studies have reported the overall effect of acupuncture on nerve recovery, motor function, sensory function, and functional recovery in SCI, the effects of acupuncture methods for SCI are yet to be analyzed [33]. At present, there are various acupuncture treatment methods for the treatment of SCI, such as EA, ear acupuncture, moxibustion, and so on. Although there is no evidence to prove which treatment is the most effective, EA is currently widely used to update the literature on EA. We conducted this systematic review to evaluate the role of EA in SCIs. Similar to previous studies, our results showed that EA can improve motor function and ADLs of patients with SCI. Moreover, we recommended GV acupoints because the spinal cord and the GV are intertwined according to the theory of traditional Chinese medicine.

### 4.3. Significance of This Review

Acupuncture, natural therapy with a long history in China, has become a treatment for insomnia with few adverse effects and permanent damage. It is guided by the theory of meridians and acupoints and is widely used for pain, joint disease, movement disorders, and SCIs. This study evaluated the effectiveness and safety of EA for the treatment of SCIs. Based on the GRADE results, there is moderate evidence that EA improves ADLs in patients with SCI. However, the evidence that EA improves sensory function in patients with SCI is low. Generally, we considered sensory function as the premise for movement, so the recovery of sensory function may increase the requirements for the use of EA for SCIs. In this study, we summarised the specific clinical points of EA treatment for SCIs. The acupoint selection was based on the GV and *Jiaji* points. The frequency of EA was five times a week, with each session lasting for 30 min, and the treatment duration was at least 2 months in most studies. In clinical research, the motor function has been reported as important for the evaluation of functional recovery. Studies have shown that the recovery of motor function in patients with SCI is based on the plasticity of corticospinal motor neurons [34]. However, the assessment of muscle tone has been explored in most studies. Among the studies included in this review, only the study by Yang et al. [30] used the modified Ashworth score to assess the improvement in muscle tone in both lower limbs. Clinically, patients with SCI have spastic paralysis with spasmodic hip adductor muscles. The important goal of SCI treatment is to relieve patients of destructive symptoms and improve their muscle strength [35, 36]. Therefore, future clinical research will focus on the use of EA to improve the muscle tone of patients with SCI.

### 4.4. Limitations of This Review

There are several limitations to our systematic review and meta-analysis. First, most of the included studies were published in the Chinese language because of the widespread application of EA in China. Second, the overall quality of the included studies was low, while most of the included studies only referred to the word “random” without performing randomization. Moreover, some studies did not explain the specific implementation method of the randomization or describe the allocation concealment and blinding methods they used. Thus, the low quality of the included studies may lead to bias towards publishing only studies with positive results [35]. Finally, very few studies included the funnel analysis. Thus, our meta-analysis could not assess publication bias. Nevertheless, our study explains how EA improves motor and sensory functions and ADLs in patients with SCI. We hope that researchers can design rigorous, large-sample, multicenter RCTs which adhere to the CONSORT guidelines. Subsequent studies should provide more reliable, evidence-based medical findings on the effectiveness of EA treatment for SCIs.

## 5. Conclusion

This review suggested that EA improved ADLs and motor function in patients with SCI, with moderate-quality evidence and improved sensory function, with low-quality evidence. These conclusions supported the use of EA to improve ADLs in patients with SCI, but the improvement of sensory function may require further research. Considering the low quality of the included studies, rigorous, large-sample, long-term clinical research is needed in the future.

## Figures and Tables

**Table 1:** Demographic characteristics of the included studies.

Included study	Sex (M/F)		Age (years)		Course of disease		Degree of injury		Segment of injury	
	EA group	Control group	EA group	Control group	EA group	Control group	EA group	Control group	EA group	Control group
Gu et al. [22]	26/6	27/3	41.2 ± 8.2	39.8 ± 7.6	28 .8 ± 11.7 d	30 .3 ± 17.6 d	ASIA-A:11, B:15, C:4, D:2, E:0	ASIA-A:9, B:16, C:3, D:2, E:0	Cervical: 4	Cervical: 2
									Thoracic: 24	Thoracic: 26
									Cauda equina: 4	Cauda equina: 2

Feng et al. [23]	13/7	15/5	38.10 ± 7.95	38.90 ± 7.08	71.85 ± 6.96 d	67.50 ± 6.41 d	ASIA-ICI:13, CI:7	ASIA-ICI:15, CI: 5	T2-T5: 2	T2-T5: 1
									T6-T12: 9	T6-T12: 10
									L1-L3: 7	L1-L3: 6
									L4-S1: 2	L4-S1: 3

Wang [24]	56/27	59/24	21–65	22–67	31–98 d	35–102 d	ASIA-motor score: 60.10 ± 4.66	ASIA-motor score: 57.35 ± 4.22	NM	NM
							ASIA-tactile score: 76.45 ± 2.69	ASIA-tactile score: 74.25 ± 4.85		
							ASIA-pain score:	ASIA-pain score:		
							74.50 ± 8.17	75.05 ± 6.66		
							ASIA-ICI:62, CI:21	ASIA-ICI: 64, CI:19		
							ASIA-motor score: 42.16 ± 9.68	ASIA-motor score: 44.23 ± 11.28		
							ASIA-tactile score: 56.76 ± 10.32	ASIA-tactile score: 58.81 ± 12.13		
							ASIA-pain score:	ASIA-pain score:		
							56.43 ± 10.25	58.49 ± 11.96		

Jiang and Chen [25]	21/9	19/11	34 ± 13	34 ± 12	3.1 ± 0.5 d	3.0 ± 0.5 d	Frankel-B:8, C:13, D:9, E:0	Frankel-B:7, C:13, D:10, E:0	C3-C4: 2	C3-C4: 2
									C4-C5: 4	C4-C5: 4
									C5-C6: 8	C5-C6: 7
									C6-C7: 9	C6-C7: 10
									C3-C5: 1	C3-C5: 1
									C4-C6: 3	C4-C6: 3
									C5-C7: 3	C5-C7: 3

Ou [26]	23/19	24/18	43.6 ± 8.7	41.2 ± 9.4	38.1 ± 16.8 d	36.7 ± 15.4 d	ASIA-B:14, C:19, D:9,	ASIA-B:13, C:21, D:8	Cervical: 10	Cervical: 11
									Thoracic: 15	Pectoral: 14
									Lumbar: 17	Lumbar: 17

Guo et al. [27]	12/18	17/13	51.80 ± 8.47	52.57 ± 7.95	6.26 ± 2.66 m	5.80 ± 2.88 m	ASIA-C:19, D:11,	ASIA-C:17, D:13	NM	NM
Li and Chi [28]	15/5	14/6	40 ± 11	45 ± 10	14–30 d	LLMSS-I:1, II:3, III:16	LLMSS-I:1, II:1, III:18	T8–T10	T8-T10	T8-T10
Deng Lao [29]	12/8	11/9	21–55	23–60	＜1 m: 3, 1–3 m: 5, 4–6 m: 12	＜1 m: 2, 1–3 m: 7, 4–6 m: 11	ASIA-motor score: 68.12 ± 5.02	ASIA-motor score: 66.23 ± 6.19	T9-L1	T9-L1
							ASIA-C and D	ASIA-C and D		

Yang et al. [30]	NM	NM	NM	ASIA-motor score: 16.02 ± 5.52	ASIA-motor score: 15.67 ± 5.21	T10-L1	T10-L1
									MAS: 0.33 ± 0.17	MAS: 0.32 ± 0.10

Wong et al. [31]	41/9	39/11	35.1 ± 13.0	34.7 ± 13.1	58.6 ± 17.1 d	57.1 ± 18.7 d	ASIA-A:28, B:22	ASIA-A:32, B:18	Quadriplegia: 19	Quadriplegia: 18
							ASIA-motor score: 41.0 ± 21.5	ASIA-motor score: 41.0 ± 17.7	Paraplegia: 31	Paraplegia: 32
							ASIA-tactile score: 63.0 ± 23.2	ASIA-tactile score: 60.8 ± 24.4		
							ASIA-pain score:	ASIA-pain score:		
							60.8 ± 27.7	59.1 ± 24.9		

Note: d, day; m, month; ICI, incomplete injury; CI, complete injury; ASIA, American Spinal Injury Association; NM, not mentioned; LLMSS, lower limb muscle strength score; MAS, modified Ashworth score.

**Table 2:** Clinical characteristics of the included studies.

Included study	Randomization method	Treatment		Intervention methods		Main points selected	Outcome indicators	Adverse events
		Duration	Frequency	EA group	Control group			
Gu et al. [22]	Randomized according to bed number	6 m	30 min, daily (7 days off every month)	EA + rehabilitation training + medication	Rehabilitation training + medication	*Jiaji* points on both sides of the injury plane	FIM	NM
Feng et al. [23]	Just mentioned random	8 w	30 min, 6 times per week	EA + rehabilitation training + medication	Rehabilitation training + medication	*Dazhui* (GV14), mingmen (GV4)	ASIA, MBI	NM
Wang [24]	Just mentioned random	3 m	NM	EA + rehabilitation training	Rehabilitation training	GV points	ASIA	NM
Jiang and Chen [25]	Random number table	80 d	30 min, daily	EA + rehabilitation training	Rehabilitation training	Cervical *jiaji* points	Frankel classification, BI, FIM	NM
Ou [26]	Random number table	8 w	30 min, 5 times per week	EA + rehabilitation training	Rehabilitation training	GV points and corresponding jiaji points on the injury plane	MMT, BI, WISCI II	NM
Guo et al. [27]	Random number table	6 w	40 min, 5 times per week	Key muscles EA + rehabilitation training	Rehabilitation training	Points on key muscles of lower limbs	MBI, MAS	NM
Li and Chi [28]	Random number table	8 w	30 min, 6 times per week	*Jiaji* EA + GV EA	Jiaji CE	T6–T11 *jiaji* points, dazhui (GV14), *mingmen* (GV4), *yanglingquan* (GB34), *zusanli* (ST36)	MBI	NM
Deng and Lao [29]	Just mentioned random	8 w	30 min, 5 times per week	*Jiaji* EA + CE + medication	CE + medication	*Jiaji* points on both sides of the injury plane	ASIA, WISCI II, SCIM	NM
Yang et al. [30]	Just mentioned random	8 w	30 min, 5 times per week	EA + weight loss walking training	Weight loss walking training	*Jiaji* points on both sides of the injury plane, *dazhui* (GV14), *mingmen* (GV4),	ASIA motor score, MBI	NM
Wong et al. [31]	Just mentioned random	12 m	30 min, 5 times per week	EA + rehabilitation training	Rehabilitation training	*Houxi* (SI3), *shenmai* (BL62), auricular acupoints	ASIA, FIM	NM

d, days; w, weeks; m, months; GV, governor vessel; CE, conventional acupuncture; ASIA, American Spinal Injury Association; FIM, functional independence measure; MMT, manual muscle testing; BI, Barthel index; MBI, modified Barthel index; WISCI II, walking index for spinal cord injury II; SCIM, spinal cord independence measure; MAS, modified Ashworth score; NM, not mentioned. Acupoint selection only indicates the main acupoints.

## Data Availability

Data are provided in supplementary information files.
