# Supplementary material for: Electroacupuncture for Spinal Cord Injury: A Systematic Review and Meta-Analysis of Randomised Controlled Trials
Source: Evid Based Complement Alternat Med. 2022 Mar 4;2022:8040555. doi: 10.1155/2022/8040555 (PMC8916891; doi:10.1155/2022/8040555)
Supplement: Supplementary Materials — Supplemental materials for this article are available online. [file 8040555.f1.zip › 8040555.f1/Supplemental Information-Figure (1).docx]

**Supplemental Information-Figure**

Supplemental information, Fig. 1 Another forest plot of difference in ASIA-tactile (when Feng et al. [23] was removed)





Supplemental information, Fig. 2. Another forest plot of difference in ASIA pain (when Feng et al. [23] was removed)





Supplemental information, Fig. 3 Another forest plot of difference in MBI/BI (when Yang et al. [30] was removed)
