# Supplementary material for: Electroacupuncture for Spinal Cord Injury: A Systematic Review and Meta-Analysis of Randomised Controlled Trials
Source: Evid Based Complement Alternat Med. 2022 Mar 4;2022:8040555. doi: 10.1155/2022/8040555 (PMC8916891; doi:10.1155/2022/8040555)
Supplement: Supplementary Materials — Supplemental materials for this article are available online. [file 8040555.f1.zip › 8040555.f1/Supplemental Information-Search strategy.docx]

**Search strategy**

1. Cochrane Library 
#1 MeSH descriptor spinal cord injury explode all trees
#2 (Spinal Cord Trauma):ti,ab,kw
#3 (Traumatic Myelopathy):ti,ab,kw
#4 (Spinal Cord Transection):ti,ab,kw
#5 (Spinal Cord Laceration):ti,ab,kw
#6 (Post-Traumatic Myelopathy):ti,ab,kw

#7 (Post Traumatic Myelopathy):ti,ab,kw

#8 (Spinal Cord Contusion):ti,ab,kw
#9 #1 or #2 or #3 or #4 or #5 or #6 or #7 or #8
#10 (#9 and (randomised and controlled and trial))
#11 MeSH descriptor Electroacupuncture explode all trees
#12 (electro-acupuncture):ti,ab,kw
#13 #11 or #12
#14 #13 and (randomised and controlled and trial)
#15 (#10 and #14)

2. Pubmed

((spinal cord injury [MeSH]) OR （spinal cord trauma）OR (traumatic myelopathy) OR (spinal cord transection) OR (spinal cord laceration) OR (post-traumatic myelopathy) OR (post traumatic myelopathy )OR (spinal cord contusion) )AND(electroacupuncture [MeSH] OR electro-acupuncture [tw])AND (randomized controlled trial [pt] OR controlled clinical trial [pt] OR randomized [tiab] OR placebo [tiab] OR clinical trials as topic [mesh: noexp] OR randomly [tiab] OR trial [ti]) NOT (animals [mh] NOT humans [mh])

3. web of science

#14 #13 AND #7

#13 #12 AND #11

#12 TS=(human*)

#11 #8 OR #9 OR #10

#10 TS=((singl* OR doubl* OR trebl* OR tripl*) SAME (blind* OR mask*))

#9 TS=(controlled clinical trial OR controlled trial OR clinical trial OR placebo)

#8 TS=(randomised OR randomized OR randomly OR random order OR random sequence OR random allocation OR randomly allocated OR at random OR randomized controlled trial)

#7 #6 AND #5

#6 TS= (electroacupuncture or electro-acupuncture or electrical acupuncture)

#5 #4 OR #3 OR #2 OR #1

#4 TS=(SCI or paraplegia* or quadriplegia* or tetraplegia*)

#3 TS=("spinal cord" and (contusion or laceration or transaction or trauma or ischemia or syndrome))

#2 TS=((spine or spinal) and (fracture* or wound* or trauma* or injur* or damage*))

#1 TS=(myelopathy and (traumatic or post-traumatic))

4. CNKI(China Journal Full-text Database): 1979.1.1-2020.9.30 
SU=('脊髓损伤'+'脊髓挫伤'+'截瘫') and SU=('电针'+'电针疗法'+'电针治疗'+'电'+'电刺激') and AB=('试验'+‘临床研究’+'前瞻性'+'随访'+'对比研究'+'多中心'+'对照'+'随机') NOT AB=('动物'+'鼠'+'兔'+'犬')

5. WanFang Date: 1990-2020

关键词:(脊髓损伤+脊髓挫伤+截瘫)*关键词：（电针+电针疗法+电针治疗+电+电刺激）*摘要:(试验+临床研究+前瞻性+随访+对比研究+多中心+对照+随机)

6. VIP: 1989-2020  
(K=('电针'+'电针疗法'+'电针治疗'+'电'+'电刺激')) AND K=('脊髓损伤'+'脊髓挫伤'+'截瘫') AND (U=('试验'+‘临床研究’+'前瞻性'+'随访'+'对比研究'+'多中心'+'对照'+'随机'))
