# Supplementary material for: Electroacupuncture for Spinal Cord Injury: A Systematic Review and Meta-Analysis of Randomised Controlled Trials
Source: Evid Based Complement Alternat Med. 2022 Mar 4;2022:8040555. doi: 10.1155/2022/8040555 (PMC8916891; doi:10.1155/2022/8040555)
Supplement: Supplementary Materials — Supplemental materials for this article are available online. [file 8040555.f1.zip › 8040555.f1/Supplemental Information-Table 1 GRADE of EA for SCI(FIM).docx]

| **EA for SCI** | | | | | | |
| --- | --- | --- | --- | --- | --- | --- |
| **Patient or population:** patients with SCI **Settings:**  **Intervention:** EA | | | | | | |
| **Outcomes** | **Illustrative comparative risks* (95% CI)** | | **Relative effect (95% CI)** | **No of Participants (studies)** | **Quality of the evidence (GRADE)** | **Comments** |
|  | Assumed risk | Corresponding risk |  |  |  |  |
|  | **Control** | **EA** |  |  |  |  |
| **FIM** |  | The mean fim in the intervention groups was **13.46 higher** (8 to 18.92 higher) |  | 222 (3 studies) | ⊕⊕⊕⊝ **moderate**^1^ |  |
| *The basis for the **assumed risk** (e.g. the median control group risk across studies) is provided in footnotes. The **corresponding risk** (and its 95% confidence interval) is based on the assumed risk in the comparison group and the **relative effect** of the intervention (and its 95% CI).  **CI:** Confidence interval; | | | | | | |
| GRADE Working Group grades of evidence **High quality:** Further research is very unlikely to change our confidence in the estimate of effect.  **Moderate quality:** Further research is likely to have an important impact on our confidence in the estimate of effect and may change the estimate. **Low quality:** Further research is very likely to have an important impact on our confidence in the estimate of effect and is likely to change the estimate. **Very low quality:** We are very uncertain about the estimate. | | | | | | |
| ^1^ No specific description of the random method, no effective blind method | | | | | | |
